# Phosphorus and cold stress: physiological and biochemical assessment in two grape (*Vitis vinifera* L.) cultivars

**DOI:** 10.1186/s40659-026-00683-0

**Published:** 2026-03-14

**Authors:** Maryam Zaeri-Behrooz, Rouhollah Karimi, Ali Khadivi, Yazgan Tunç

**Affiliations:** 1 Grape Production and Genetic Improvement Department, Iranian Grape and Raisin Institute, Malayer University, Malayer, Iran; 2 Department of Horticulture and Landscape Engineering, Faculty of Agriculture, Malayer University, Malayer, Iran; 3 Department of Horticultural Sciences, Faculty of Agriculture and Natural Resources, Arak University, Arak, 38156-8-8349 Iran; 4 Republic of Türkiye, Ministry of Agriculture and Forestry, Hatay Olive Research Institute Directorate, General Directorate of Agricultural Research and Policies, Hassa Station, Hassa, Hatay 31700 Türkiye

**Keywords:** Chilling tolerance, Lipid peroxidation, Nutrition, Polyamines, Soluble sugars

## Abstract

Low temperature is one of the most damaging factors in the production of horticultural crops. This study was conducted to investigate the mechanisms of frost tolerance resulting from the use of different concentrations of phosphorus (P) fertilizer in two grape (*Vitis vinifera* L.) cultivars, ‘Khalili’ and ‘Perlette’, factorially based on a completely randomized design on potted vines. The cultivars were treated with a Hoagland nutrient solution containing three different P concentrations (0, 0.5, and 1 mM), and were examined at two temperatures, 24 °C and 2 °C. The results showed that cold tolerance was higher in the ‘Khalili’ cultivar compared with the ‘Perlette’ cultivar. In both cultivars, the samples treated with 1 mM P concentration exhibited the lowest values for cold tolerance-related indices. The highest cold tolerance, measured using the electrolyte leakage method, was observed in the ‘Khalili’ cultivar at 0.5 mM P concentration. The highest amounts of hydrogen peroxide and malondialdehyde were observed at 0 and 1 mM P concentrations under the + 2 °C temperature treatment. The lowest activity of antioxidant enzymes, soluble sugars, soluble proteins, and polyamines was observed at a 1 mM P concentration in both ‘Khalili’ and ‘Perlette’ cultivars. In this treatment, the lowest amounts of chlorophyll, anthocyanins, and leaf calcium, magnesium, zinc, copper, and active iron were observed. The ratio of active iron to total iron significantly decreased at the 1 mM P concentration, and leaf chlorosis was visible in this treatment. These results indicate that optimum concentrations of P (0.5 mM) can physiologically and biochemically lead to improved cold tolerance of grapevine plants.

**Supplementary Information:**

The online version contains supplementary material available at 10.1186/s40659-026-00683-0.

## Introduction

Grapes (*Vitis vinifera* L.) are one of the important fruits in temperate regions, which, in addition to fresh consumption, have many health-beneficial by-products. Frost is one of the most important factors that reduce the yield of vineyards located in temperate climates, which in some years causes large economic losses to grape growers. Therefore, the use of cold stress reduction methods is one of the key requirements in the management of vineyards and the sustainable production of grapes in susceptible regions with cold weather conditions [30].

Under cold stress, certain physiological and biochemical changes occur in the plant, such as the reduction of tissue relative water content (RWC), the accumulation of compatible solutions such as soluble carbohydrates, amino acids (proline, betaine-glycine), antifreeze proteins and the phytohormone abscisic acid [12, 28, 45]. These changes reduce the freezing point by protecting the cell structure against dehydration caused by freezing. During cold stress, by binding to phospholipids and creating hydrogen bonds with membrane proteins, sugars stabilize cells in the face of low temperatures, thus reducing water loss and maintaining cell turgor in these conditions [17, 45]. Also, sugars protect against cold by reducing the freezing point of cell sap [28]. Under cold stress conditions, oxidative stress usually occurs due to changes in the balance between light received and light absorption efficiency. By increasing the leakage of electrons to oxygen molecules, oxidative stress facilitates the formation of reactive oxygen species (ROS) such as superoxide (O^− 2^), hydrogen peroxide (H_2_O_2_), hydroxyl radicals (OH^−^), and singlet oxygen (O^−^; [16]). To reduce or prevent oxidative stress damage caused by low temperatures, plants are equipped with enzymatic systems such as superoxide dismutase, catalase (CAT), guaiacol peroxidase (GPX), and ascorbate peroxidase (APX) and non-enzymatic (phenols and flavonoids) antioxidant systems, which protect the photosynthetic apparatus against oxidative stress injuries in most plant species ([31, 32, 36, 37].

So far, various methods such as covering the vines, selecting suitable cultivars, vineyard climate management, and vine nutrition management have been used to reduce frost damage in fruit trees [12, 28, 47]. Although cold tolerance strongly depends on cultivar genetics [29, 30], the correct nutrition is particularly important for improving low-temperature resistance of plants [36, 59].

Phosphorus (P) is one of the elements that often limit agricultural production worldwide. In the soil-plant system, P has very little mobility in soil because it is strongly absorbed by iron (Fe) and aluminum oxides in heavily weathered soils and by forming complexes with calcium (Ca) in calcareous soils [15]. Physiologically, P is involved in the structure of macromolecules of ATP, DNA, RNA, cell membrane phospholipids, carotenoids, and gibberellin hormone, and also many metabolic reactions (i.e., photophosphorylation and respiration) are dependent on P concentration [10]. The presence of P is important for the function of aquaporin proteins that play a role in the movement of water in the xylem. In addition, inorganic pyrophosphate, a byproduct of starch production, is essential for the function of the phloem [38].

Plants show a series of morphological, physiological, and biochemical responses during exposure to P deficiency. The modification of root architecture is one of the most important morphological changes, which causes the formation of secondary and hairy roots near the soil surface, affecting the absorption of other nutrients [43, 46]. Also, during the P deficiency, the plant will show a decrease in root meristem growth and a reduction in root growth. When a plant is exposed to P deficiency, a series of physiological changes such as secretion of acid phosphatase, carboxylate, RNases, and protons occur [27]. In this condition, the increasing sucrose supply to roots acts as a carbon source and also as a signal to initiate changes in the expression of metabolic genes and root development [22]. Under P-deficiency conditions, the starch content increases in the cell. If the mineral phosphate level increases too much, the starch content in the cell will decrease [2]. Another important change during P deficiency is the change in the structure of the plasma membrane of the cell, where phospholipids of the membrane are replaced by sulfolipids [39].

Although many studies have been reported on the effect of nutritional elements on frost tolerance in grapes or other trees, there are limited reports regarding the effect of P on the cold hardiness of plants. In a study, the use of calcium chloride on ten-year-old ‘Thompson Seedless’ grapes increased soluble carbohydrates and proteins and increased cold resistance [21]. In ‘Sultana’ grapes, an increase in spring cold resistance was observed with foliar application of calcium sulfate combined with zinc sulfate [29]. In citrus fruits, P deficiency under normal conditions and without stress caused an increase in CAT and APX and a decrease in GPX [44]. On the other hand, some reports show that nutrients can increase these metabolites even in stressful conditions [32].

This research was conducted to investigate the effect of P nutrition at different concentrations to improve the cold tolerance of two grape cultivars. So far, the effect of applying P concentrations on the cold tolerance of grapes has not been investigated, while low or high P levels can affect the processes related to cold acclimation in plants by affecting the metabolic activity of the plant. We hypothesize that moderate P levels (0.5 mM) increase cold tolerance, while excessive P decreases this tolerance due to mineral imbalance. Therefore, this research aimed to investigate the effect of feeding different concentrations of P on physiological and biochemical indices related to cold tolerance in two grape varieties with different cold tolerance.

## Materials and methods

This research was carried out on potted vines of two grape varieties factorially (3 × 2 × 2) based on a completely randomized design with 6 replicates (2 pots per replicate) in the research greenhouse of Malayer University. The first factor included P supply at three concentrations at 0, 0.5 and 1.0 mM, the second factor included two grape varieties, ‘Perlette’ (as a cold-sensitive) and ‘Khalili’ (as a cold tolerant, [30]), and the third factor included two temperature regimes, including 24 °C (as a normal temperature) and 2 °C (as a chilling temperature). The vines were grown in 7-liter pots of hydroponic culture medium mixture, including perlite and cocopeat (in equal volume ratio), under greenhouse conditions (24 ± 2 °C). The pots were fed with Hoagland’s solution three times a week and watered once with distilled water to prevent the salinity of the culture medium. It should be noted that the composition of Hoagland’s solution (apart from P) remained the same throughout the experiment.

After 4 months, just when the vines reached the stage of 20 to 25 leaves, half of the vines were exposed to low temperature (2 °C) for 12 h in the cooling chamber (Rad Electronic, Iran) with a light intensity of 200 µmol m^− 2^ s^− 1^ during the night. The starting temperature of the cold treatment in the cold chamber was considered based on the approximate temperature of the environment at the time of moving the pots (15 °C), and the rate of decrease in the temperature of the chamber was 2 °C per hour. After the cold room reached 2 °C, the vines were kept at this temperature for 12 h. After this period, the temperature of the cooling chamber was gradually increased, and within 5 h it reached ambient temperature. Next, the low-temperature-treated vines were removed from the cooling chamber and placed in the greenhouse for three days to recover. Then, the fully developed leaves of the vines were used to measure the following physiological and biochemical indices.

Leaf chlorophyll content (SPAD index) was measured from the middle leaves of canes around the vine using a SPAD-CL01 (Atago, Japan) chlorophyll meter. The amount of electrolyte leakage (EL) of the leaf tissue was measured using an electrical conductivity meter (Atago, Japan) in two stages, before autoclaving (EC_1_) and after (EC_2_). The amount of EL was calculated through the relation EL = (EC_1_/EC_2_) ×100 [12].

Membrane lipid peroxidation was measured by the thiobarbituric acid test (TBAT) through measuring the malondialdehyde [23]. Leaf H_2_O_2_ was measured according to Loreto and Velikova [42], and its content was calculated by comparing their absorbance at the wavelength of 390 nm and its standard curve in the range from 100 to 1000 µmol ml^− 1^.

Leaf proline was assayed based on the colorimetric ninhydrin method, quantitatively measured at a wavelength of 518 nm in a spectrophotometer (Spekol 2000 model, Germany), and expressed in µmol g^− 1^ fresh weight [3]. Soluble protein was measured with a brilliant blue reagent (G-250) at a wavelength of 595 nm [6]. Using a standard curve prepared from different concentrations of bovine albumin, the concentration of soluble protein was expressed in terms of mg g^− 1^ FW.

To measure the activity of antioxidant enzymes, fresh leaf samples (200 mg) were ground and powdered in liquid nitrogen and kept at -80 °C until the time of measurement. The activities of GPX [25], CAT [5], and APX [49] enzymes were measured at wavelengths of 465, 240, and 290 nm, respectively. Each unit of activity of GPX and CAT was considered as the amount of these enzymes that cause the reduction of one µmol of H_2_O_2_ per minute. Each unit of APX enzyme activity was considered as the amount of enzyme that oxidizes one µmol of APX per minute. The activities of all three enzymes were expressed as units per mg of leaf protein.

Leaf RWC was determined based on [11] by weighing the leaf pieces (with 1 cm radius) before (FW) and after oven drying (48 h, 72 ◦C; dry weight), and also after immersing in distilled water for 24 h (turgid weight). The leaf RWC was calculated through the relation RWC = (FW-DW)/(TW-DW) × 100.

Soluble sugars were measured according to the method of Shin et al., [54] by injecting 10 µL of ethanolic leaf extract into the HPLC device (Unicam-Crystal-200 model, England). The used Spherisorb C_8_-ODS_2_ column was 150 mm long and 4.6 mm in diameter, and the particle diameter was 0.3µ. The mobile phase consisted of sodium citrate buffer, pH = 5.5, and ultrapure acetonitrile with a ratio of 99:1 and a flow rate of 0.1 ml min^− 1^. Based on the inhibition time and using glucose, fructose, and sucrose standards, the type and amount of soluble sugars in unknown samples were determined and expressed as µmol g^− 1^ FW [54].

For extraction of polyamines (putrescine, spermidine, and spermine), a volume of 2 ml HClO_4_ (4%) in comprising 1, 7 diaminoheptane-2HCl were added to the grounded tissues of frozen leaves (250 mg) and placed for 60 min at 4 °C. The homogenates were filtered through a Whatman filter (0.45 µ). One ml of dansyl chloride solution (10 mg ml^− 1^ acetone) and 1 ml of carbonate buffer (pH 9) were added to 0.2 ml one the homogenates and then heated for 60 min at 60 °C. For extraction of dansylated polyamines, toluene (3 ml) was added to homogenate. To measure the concentration of polyamines, 10 µL of the final solution of the separation step was injected into the reverse-phase HPLC device with a small column with a length of 10 cm and an inner diameter of 3 mm, type Chorompack-Nederland. The mobile phase consists of a mixture of ultra-pure acetonitrile and deionized water with a ratio of 72 to 28 (V/V), respectively, which is moved at a speed of 2 ml/min with an isocratic system. The detector of the device was UV type and set at a wavelength of 337 nm [57].

To measure ABA, an HPLC device equipped with a UV-Vis SPD MLOAD detector of Photodiode array type and a Diamonsic-C18 column (5 μm particle diameters, 250 mm × 4.6 mm/day) was used. The mobile phase consisted of 20 to 75% methanol in 1% acetic acid with a flow rate of 1.2 ml min^− 1^. Based on the inhibition time according to the ABA standard sample and the area under the curve, the amount of ABA in the samples was determined at a wavelength of 260 nm and expressed as ng g^− 1^ FW [41].

For the starch assay, 1 g of leaf samples was homogenized in 5 mL of 80% ethanol. The mixture was centrifuged at 5800 rpm, and the precipitate was washed three times with 80% ethanol. The remaining precipitates were dried, and 5 mL of water and 6.5 mL of 52% perchloric acid were added. The test tubes were kept at 4 °C for 15 min, and then centrifuged again. The liquid phase was transferred to test tubes and stored in ice. After adding 0.67 mL of perchloric acid to the test tubes, extraction was repeated, and the samples were centrifuged for a third time. At this stage, the collected liquid phase was separated, and the volume was adjusted to 100 mL with distilled water. From this solution, 0.2 mL was taken and mixed with 3 mL of anthrone, and the test tubes were placed in a water bath at 90 °C for 10 min. After this time, the test tubes were quickly cooled in ice water, and the absorbance of the samples was read using a spectrophotometer at a wavelength of 630 nm [24].

To measure leaf anthocyanin, 0.2 g of fresh plant sample was ground with 3 mL of acidic methanol (a mixture of methanol and HCl in a 99:1 ratio). The samples were centrifuged at 10,000 rpm for 15 min. The absorbance of the samples was then measured using a spectrophotometer at wavelengths of 535 and 653 nm. The absorbance at 653 nm was subtracted to remove the chlorophyll effect from the main anthocyanin absorption (which has its highest absorption at 535 nm), and the absorbance difference of the samples was reported [48].

To measure the leaf nutrient content, the middle and mature leaves were washed with distilled water, and then the samples were dried at 75 °C for 72 h. The concentrations of nitrogen (N), P, K, Ca, Magnesium (Mg), Zn, and copper (Cu) were evaluated separately. The extraction was performed using the wet digestion method [33]. For this purpose, one gram of leaf powder was added to 10 mL of concentrated nitric acid (65%), and the mixture was placed in a water bath at 65 °C for two hours. Then, 2.6 mL of 20% H_2_O_2_ was added to the samples. The samples were filtered using Whatman 42 filter paper, and their volume was adjusted to 50 mL with distilled water. K was measured using a flame photometer (G 405, Germany), Mg, Ca, Fe, Zn, Mn, and Cu were determined using an atomic absorption spectrophotometer (AANALYST70 model, USA), N using the Kjeldahl method, and P was assayed using a spectrophotometer at 470 nm [33].

For the measurement of active Fe, the leaf sample was dried in an oven at 72 °C for 48 h and then ground. One gram of the ground sample was placed in a test tube, and 100 mL of 1 N HCl was added, then shaken for 16 h. After this step, the solution was filtered through the Whatman 42 filter paper and analyzed using an atomic absorption spectrometer [56].

Data analysis was performed using the SAS 9.4 statistical software (GLM procedure), and mean comparisons were conducted using Duncan’s multiple range test at a 1% probability level. Duncan’s test compares treatments independently of each other. This test compares all treatments two by two and is more powerful than the LSD test. In some cases, it better shows the significance between the means.

## Results

### Leaf EL and RWC

The percentage of EL was significantly affected by the main effects of P concentration, cultivar, and temperature (*p* ≤ 0.01), as well as the interaction effects of concentration, cultivar, and temperature (*p* ≤ 0.05). In ‘Khalili’, the lowest EL percentage was found following 0.5 mM P and + 24 °C treatments. However, in ‘Perlette’, the highest EL percentage was found following in non-P-treated plants (P at 0 mM) under + 2 °C. The RWC was also significantly affected by the main effects of P concentration, cultivar, and temperature (*p* ≤ 0.01), and the interaction effects of concentration and temperature (*p* ≤ 0.05). A decrease in temperature caused a reduction in the RWC, with the reduction being more pronounced in the ‘Perlette’ than the ‘Khalili’ cultivar. The highest RWC was observed with 0.5 mM P concentration under + 24 °C in both ‘Khalili’ and ‘Perlette’ cultivars, and no significant difference was found between P at 0 and 1 mM concentrations (Table 1).

**Table 1:** The interaction effect of phosphorus (P; 0, 0.5, and 1.0 mM) and temperatures (+ 2 and + 24 °C) on some physiological and biochemical indices of leaves in two grape cultivars differing in cold tolerance

Treatment	Electrolyte leakage (%)	Relative water content (%)	Chlorophyll (SPAD)	Anthocyanin (mg g^− 1^ FW)	Soluble protein (mg g^− 1^ FW)
**Phosphorus concentration (PC; mM)**					
0	50.3 ± 3.7 a	63.4 ± 2.17 b	7.3 ± 0.43 a	21.8 ± 0.43a	6.9 ± 0.17 b
0.5	28.3 ± 3.4 c	80.6 ± 1.23 a	5.9 ± 0.46 b	19.6 ± 2.3 b	8.9 ± 0.21 a
1.0	31.1 ± 3.01 b	58.2 ± 1.09 c	2.4 ± 0.29 c	18.8 ± 0.56 c	3.3 ± 0.21 c
*P*_(PC)_	*	*	*	*	*
**Grape cultivar (GC)**					
Khalili (K)	32.6 ± 2.5 b	73.3 ± 1.12 a	5.1 ± 0.44 a	14.32 ± 1.8 a	6.9 ± 0.44 a
Perlette (P)	40.5 ± 2.9 a	64.6 ± 1.13 b	5.3 ± 0.45 a	14.5 ± 1.8 a	5.8 ± 0.42 b
*P*_(GC)_	*	*	ns	ns	*
**Temperature treatment (TT)**					
+ 24 °C (Normal; N)	25.3 ± 1.9 b	77.1 ± 1.78 a	3.8 ± 0.1 a	11.4 ± 1.9 a	6.3 ± 0.35 a
+ 2 °C (Stress; S)	47 ± 1.9 a	60.5 ± 1.95 b	6.5 ± 0.1 b	17.4 ± 1.20 b	6.4 ± 0.51 a
*P*_(TT)_	*	*	*	*	ns
**P concentration × Cultivar × Temperature**					
0 × K × N	31.4 ± 0.53 e	76.7 ± 1.19 c	8.7 ± 0.18 a	20.03 ± 0.08 c	7.5 ± 0.0.2 bc
0 × K × S	56.8 ± 0.54 b	62.3 ± 2.51 f	5.5 ± 0.09 c	23.7 ± 0.22 b	7.5 ± 0.22 bc
0.5 × K × N	14.5 ± 0.74 g	83.3 ± 1.88 a	7.1 ± 0.24 b	11.23 ± 0.30 b	8.9 ± 0.26 ab
0.5 × K × S	35.5 ± 0.74 d	70.7 ± 1.16 d	4.3 ± 0.08 d	27.2 ± 0.27 a	9.9 ± 0.03 a
1.0 × K × N	19.4 ± 0.71 f	79.8 ± 2.10 bc	3.4 ± 0.08 d	2.13 ± 0.21e	4.4 ± 0.21 d
1.0 × K × S	37.9 ± 0.56 d	57.3 ± 2.28 fg	1.3 ± 0.09 e	1.6 ± 0.096 e	3.1 ± 0.03 ed
0 × P × N	46.8 ± 0.54 c	62.9 ± 1.26 f	8.8 ± 0.27 a	20.7 ± 0.44 c	6.3 ± 0.35 c
0 × P × S	66.5 ± 0.53 a	51.4 ± 2.35 g	6.1 ± 0.25 c	22.9 ± 0.50 b	6.4 ± 0.35 c
0.5 × P × N	19.8 ± 0.74 f	81.6 ± 2.72 ab	7.8 ± 0.32 ab	11.8 ± 0.72 d	7.9 ± 0.32 bc
0.5 × P × S	43.8 ± 0.55 c	64.7 ± 2.02 ef	4.3 ± 0.24 d	28.1 ± 0.65 a	9.1 ± 0.30 ab
1.0 × P × N	20.5 ± 0.74 f	68.3 ± 2.23 de	3.4 ± 0.15 d	2.5 ± 0.48 e	3.3 ± 0.01 ed
1.0 × P × S	46.1 ± 0.56 c	54.5 ± 1.99 fg	1.56 ± 0.16 e	1.2 ± 0.13 e	2.7 ± 0.12 e
*P*_(PC × GC)_	*	ns	ns	ns	ns
*P*_(PC × TT)_	ns	ns	**	*	*
*P*_(GC × TT)_	ns	ns	ns	ns	ns
*P*_(PC × GC × TT)_	*	ns	ns	ns	ns

Mean values marked with the different letters are significantly different (*p* ≤ 0.05) using Duncan’s multiple range. Means ± SE (*N* = 3)

### Leaf chlorophyll, anthocyanin, and soluble protein

The amounts of leaf chlorophyll (measured as SPAD) and anthocyanin were significantly influenced by the main effects of P concentration and temperature (*p* ≤ 0.01), as well as the interaction effect of P concentration and temperature (*p* ≤ 0.05). Overall, an increase in P concentration and a decrease in temperature led to a reduction in chlorophyll content. The highest chlorophyll content was observed with P untreated samples under + 24 °C in both ‘Khalili’ and ‘Perlette’ cultivars, while the lowest chlorophyll content was found in plants treated with 1 mM P under + 2 °C in both cultivars. With second level of P and decrease in temperature, anthocyanin content increased. The highest anthocyanin content was observed in plants treated with 0.5 mM P under + 2 °C in both ‘Khalili’ and ‘Perlette’ cultivars, while the lowest anthocyanin content was found with 1 mM P concentration under both + 24 °C and + 2 °C. Protein content was significantly affected by the main effects of P nutrition and cultivar (*p* ≤ 0.01), and the interaction effect of P concentration and temperature (*p* ≤ 0.05) was also significant. The highest protein content was found following 0.5 mM P and + 2 °C treatments in both cultivars, while the lowest protein content was found in plants treated with 1 mM P under both + 24 °C and + 2 °C (Table 1).

### Leaf MDA and H_2_O_2_

**Fig. 1:**
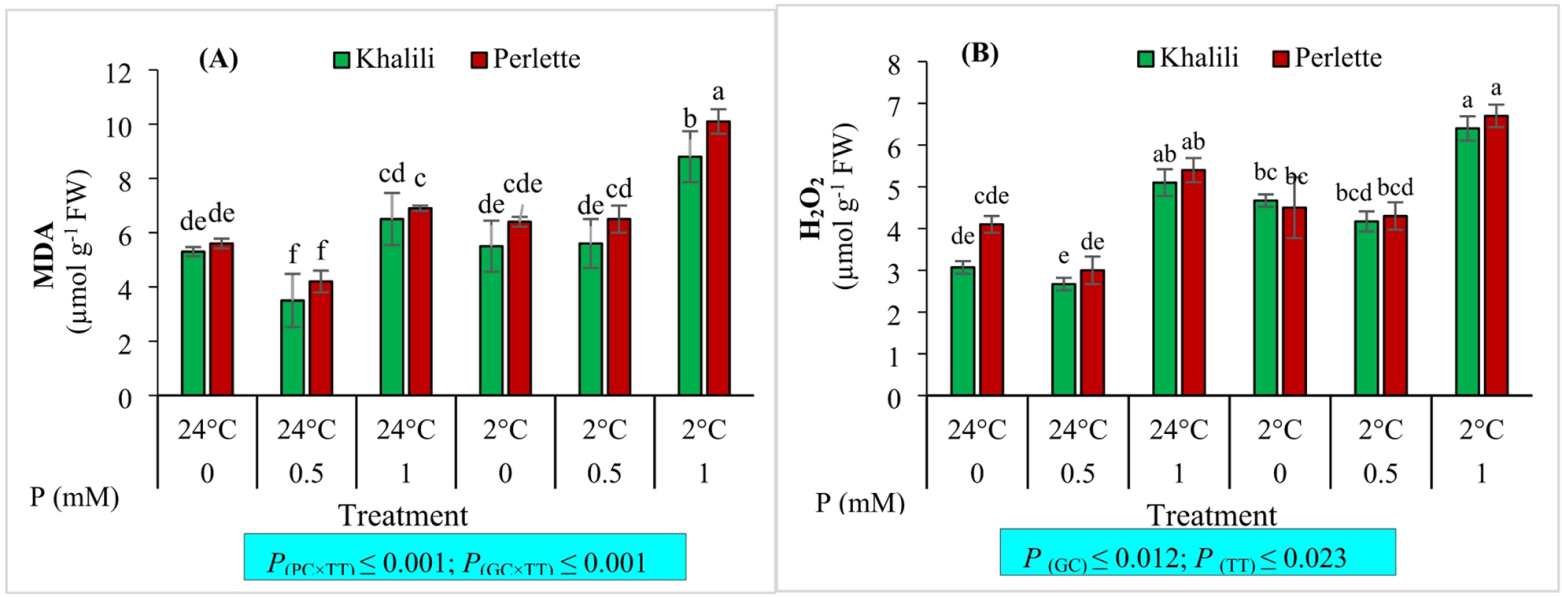
The interaction effect of phosphorus (P; 0, 0.5, 1.0 mM) and temperatures (+ 2 and + 24 °C) on malondialdehyde (MDA; **A**) and hydrogen peroxide (H_2_O_2_; **B)** content of leaves in two grape cultivars differing in cold tolerance. Mean values marked with the different letters are significantly different (*p* ≤ 0.05) using Duncan’s multiple range. Means ± SE (*N* = 3). *P*_(GC)_, effect of grape cultivar (GC); *P*_(TT)_, effect of temperature treatment (TT); *P*_(PC×TT)_, interaction effect of phosphorous concentration (PC) and TT; *P*_(GC×TT)_, interaction effect of GC and TT

The level of MDA was significantly affected by the main effects of P concentration, cultivar, and temperature (*p* ≤ 0.01), as well as by the interaction effects of concentration and temperature, and concentration and cultivar (*p* ≤ 0.05). The highest MDA level was observed in vines fertilized with 1 mM P under + 2 °C in the ‘Perlette’ cultivar. In comparison, the lowest MDA level was recorded with 0.5 mM P under + 24 °C in both the ‘Khalili’ and ‘Perlette’ cultivars. The level of H_2_O_2_ was also significantly affected by P concentration and temperature (*p* ≤ 0.01). The highest H_2_O_2_ level was observed with 1 mM P concentration under + 2 °C in both the ‘Perlette’ and ‘Khalili’ cultivars. In comparison, the lowest level was recorded with 0.5 mM P under + 24 °C in the ‘Khalili’ cultivar (Fig. 1).

### Leaf polyamines and proline

**Fig. 2:**
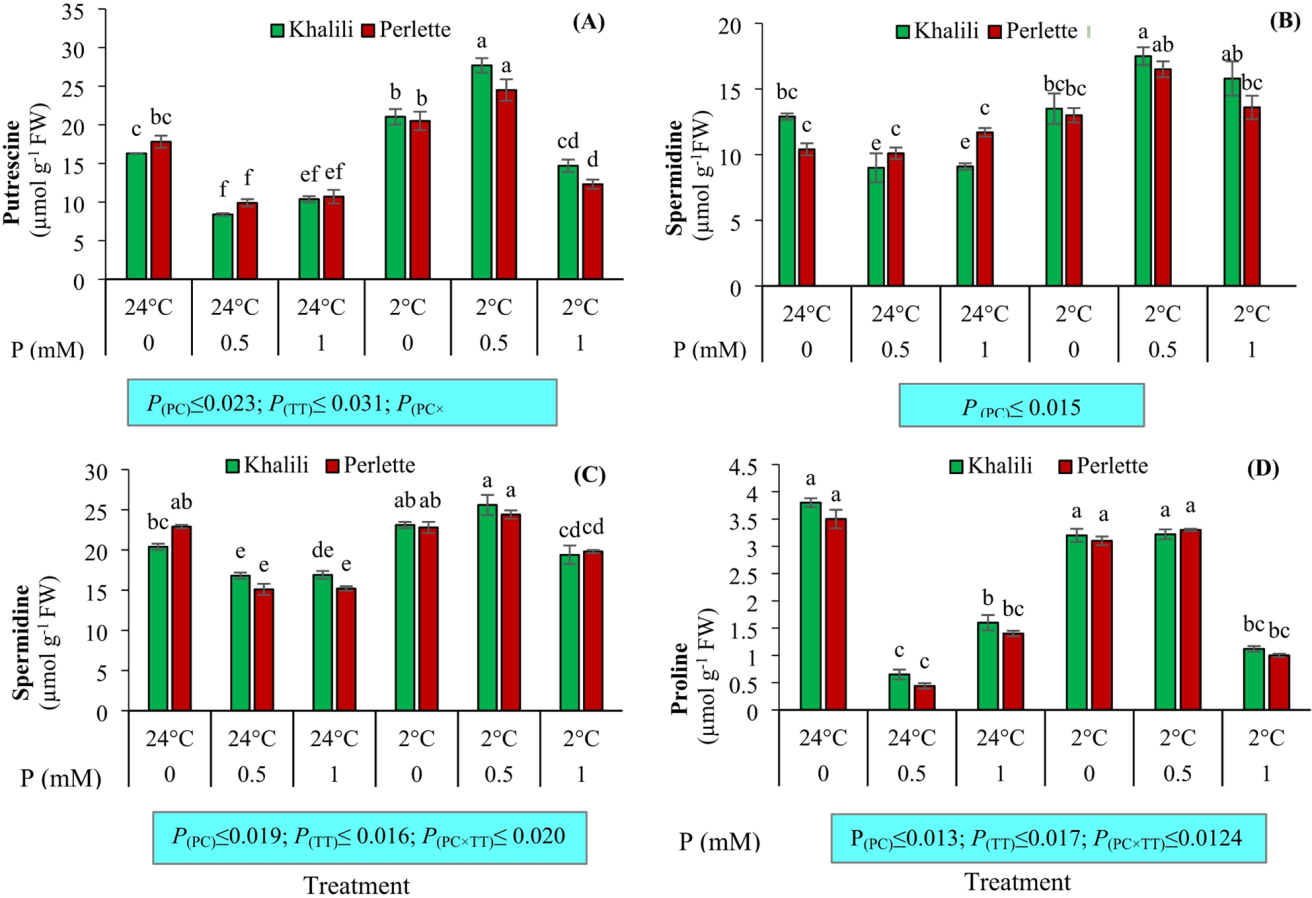
The interaction effect of phosphorus (P; 0, 0.5, 1.0 mM) and temperatures (+ 2 and + 24 °C) on putrescine (**A**), spermine (**B**), spermidine (**C**), and proline (**D**) content of leaves in two grape cultivars differing in cold tolerance. Mean values marked with the different letters are significantly different (*p* ≤ 0.05) using Duncan’s multiple range. Means ± SE (*N* = 3). *P*_(PC)_, effect of phosphorous concentration (PC); *P*_(TT)_, effect of temperature treatment (TT); *P*_(PC×TT)_, interaction effect of PC and TT

The polyamines putrescine and spermine were significantly influenced by P concentration, temperature, and their interaction effects (*p* ≤ 0.01). With 0.5 mM P and + 2 °C treatments, both ‘Khalili’ and ‘Perlette’ cultivars showed the highest putrescine levels, while the lowest levels were observed under + 24 °C. The highest spermine levels were also recorded with 0 and 0.5 mM P-fertilized vines under + 2 °C in both cultivars. The lowest spermine levels were observed in vines supplied with 0.5 mM P under + 24 °C. Spermidine showed significant changes (*p* ≤ 0.01) only under temperature, with the highest levels observed in cold stress treatments. The proline content was also significantly affected by P concentration, temperature, and their interaction effects (*p* ≤ 0.01). Proline increased in P-untreated vines kept at ambient temperature (+ 24 °C) and also in those plants under low temperature stress, with the greatest increases found with 0.5 mM P (Fig. 2).

### Leaf soluble sugars and starch

**Fig. 3:**
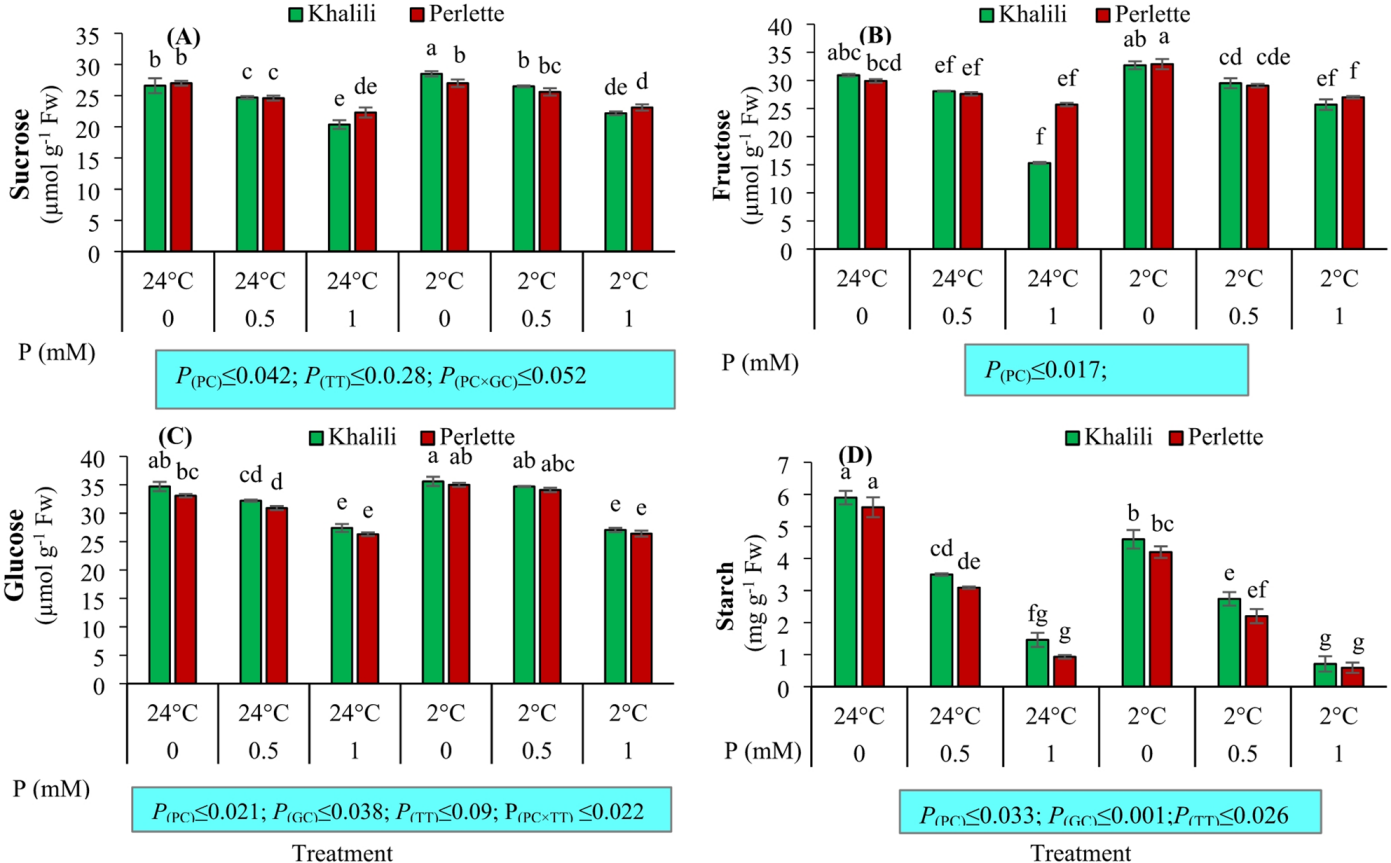
The interaction effect of phosphorus (P; 0, 0.5, 1.0 mM) and temperatures (+ 2 and + 24 °C) on sucrose (**A**), fructose (**B**), glucose (**C**), and starch (**D**) content of leaves in two grape cultivars differing in cold tolerance. Mean values marked with the different letters are significantly different (*p* ≤ 0.05) using Duncan’s multiple range. Means ± SE (*N* = 3). *P*_(PC)_, effect of phosphorous concentration (PC); *P*_(GC)_, effect of grape cultivar (GC); *P*_(TT)_, effect of temperature treatment (TT); *P*_(PC×TT)_, interaction effect of PC and TT; *P*_(GC×PC)_, interaction effect of GC and PC

The levels of sucrose and fructose were significantly influenced by the main effects of P concentration, cultivar, temperature, and the interaction effects of concentration and temperature (*p* ≤ 0.01). The glucose level was also significantly affected by the three main factors: P concentration, temperature, and their interaction effects (*p* ≤ 0.01). The highest sucrose and fructose contents were observed in vines treated with + 2 °C in both the ‘Khalili’ and ‘alone in Perlette’, while the lowest levels were recorded in vines supplied with P at 1 mM under both temperature treatments. The highest glucose level was observed with non-P-treated vines under + 2 °C in the ‘Khalili’ cultivar, while the lowest level was with 1 mM P under both temperature treatments. The starch level was also significantly affected by the main effects of P concentration and temperature (*p* ≤ 0.01) and cultivar (*p* ≤ 0.05). The lowest starch content was found following 1 mM P treatments under both temperature treatments, while the highest content was recorded with non-P-treated vines under + 24 °C in both cultivars (Fig. 3).

### Leaf antioxidant enzyme activity

**Fig. 4:**
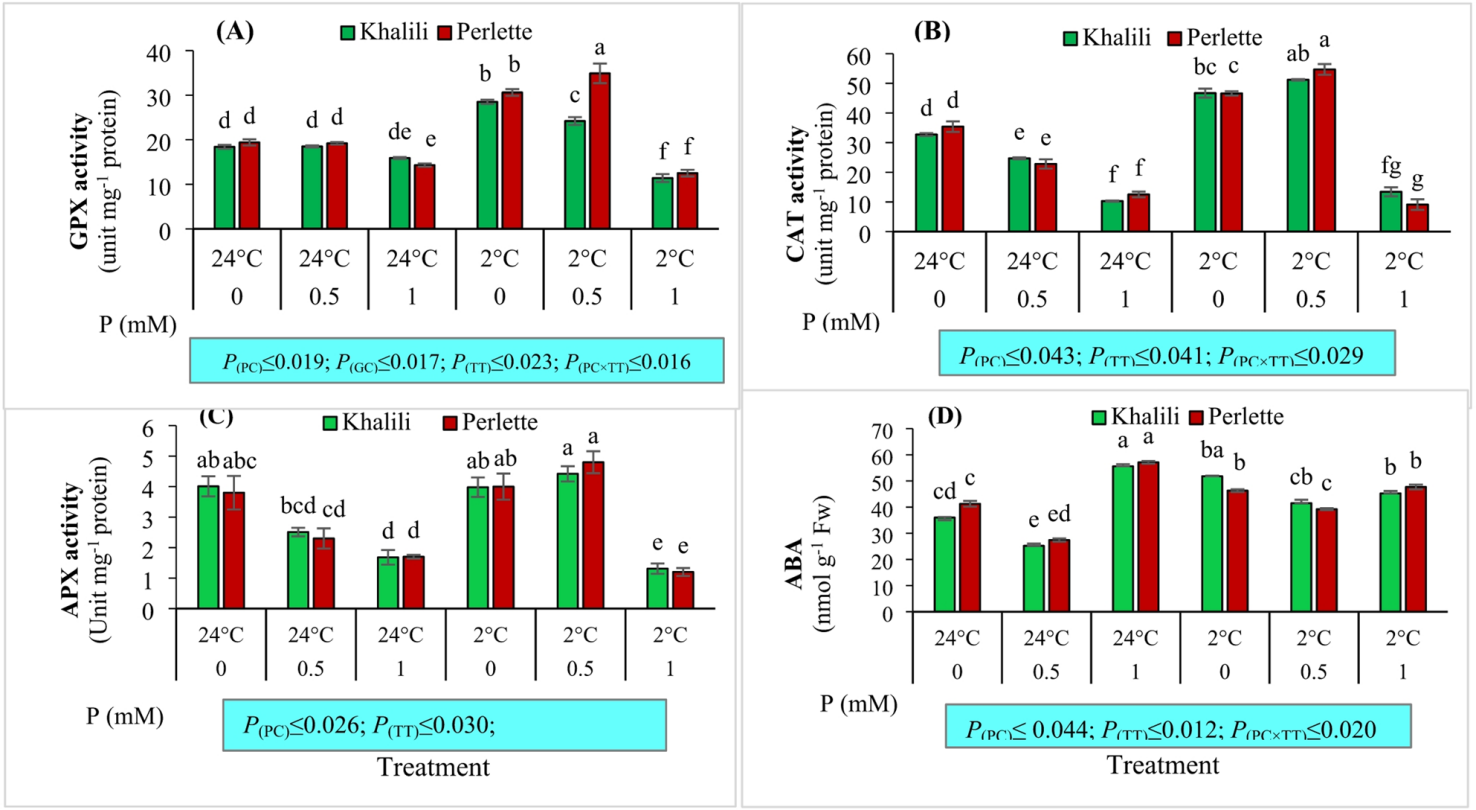
The interaction effect of phosphorus (P; 0, 0.5, 1.0 mM) and temperatures (+ 2 and + 24 °C) on guaiacol peroxidase (**A**; GPX), catalase (CAT; **B**), ascorbate peroxidase (APX; **C**), and abscisic acid activity and abscisic acid (ABA; **D**) content of leaves in two grape cultivars differing in cold tolerance. Mean values marked with the different letters are significantly different (*p* ≤ 0.05) using Duncan’s multiple range. Means ± SE (*N* = 3). *P*_(PC)_, effect of phosphorous concentration (PC); *P*_(GC)_, effect of grape cultivar (GC); *P*_(TT)_, effect of temperature treatment (TT); *P*_(PC×TT)_, interaction effect of PC and TT

The activity of the enzymes CAT, GPX, and APX was significantly influenced by the main effects of P concentration and temperature, as well as the interaction effect of P concentration and temperature (*p* ≤ 0.01). Vines treated with 1 mM P under temperature treatments (+ 24 °C and + 2 °C) showed the lowest enzyme activities in both ‘Khalili’ and ‘Perlette’ cultivars. The activity of GPX was also significantly affected by the interaction effects of P concentration, cultivar, and temperature (*p* ≤ 0.01). The highest activity of this enzyme was observed with 0.5 mM P and + 2 °C treatments in the ‘Perlette’ cultivar. In comparison, the lowest activity was recorded with 1 mM P concentration and + 2 °C treatments in the ‘Khalili’ cultivar (Fig. 4).

### Leaf ABA

The ABA content was significantly affected by the main factors of P concentration and temperature (*p* ≤ 0.01). In addition, in the interaction effects, the ABA hormone was significantly influenced by the combination of temperature and concentration (*p* ≤ 0.05). As P concentration increased and temperature decreased, the ABA content increased, such that the highest concentration of ABA was observed with 1 mM P concentration and + 2 °C in both ‘Khalili’ and ‘Perlette’ cultivars, while the lowest level of this hormone was recorded with 0.5 mM P and + 24 °C treatments in both cultivars (Fig. 4).

### Leaf macronutrients

The contents of N, K, P, and Ca were significantly influenced by the main effects of P concentration, cultivar, and temperature (*p* ≤ 0.01). However, the Mg content was only significantly affected by the main effect of P concentration. Among the interaction effects, only K was significantly influenced by the interaction of temperature and cultivar (*p* ≤ 0.05). As the P concentration in the nutrient solution increased, the levels of N, K, and P increased. The highest levels of these elements were observed with 1 mM P concentration and + 24 °C in the ‘Khalili’ cultivar. In comparison, the lowest levels were recorded at a 0 mM P concentration and + 2 °C in the ‘Perlette’ cultivar (Table 2). The highest Ca concentration was observed with 0.5 mM P concentration and + 2 °C in the ‘Khalili’ cultivar, while the lowest concentration was found at a 1 mM P concentration and + 24 °C in the ‘Perlette’ cultivar. The highest and lowest Mg concentrations were observed with 0 mM P concentration and + 24 °C and + 2 °C, as well as with 1 mM P concentration and + 2 °C in both ‘Khalili’ and ‘Perlette’ cultivars (Table 2).

**Table 2:** The interaction effect of phosphorus (P; 0, 0.5, 1.0 mM) and temperatures (+ 2 and + 24 °C) on macronutrient content of leaves in two grape cultivars differing in cold tolerance

Treatment	Nitrogen (mg/g)	Potassium (mg/g)	Phosphorus (mg/g)	Calcium (mg/g)	Magnesium (mg/g)
Phosphorus concentration (PC; mM)					
0	24.8 ± 3.2 b	16.3 ± 0.8 b	0.43 ± 0.04 c	29.2 ± 0.85 b	2.68 ± 0.03 a
0.5	31.6 ± 1.9 a	22.7 ± 0.49 a	1.41 ± 0.0 b	31.7 ± 0.83 a	1.7 ± 0.0 b
1.0	31 ± 2.9 a	22.3 ± 0.74 a	1.57 ± 0.04 a	20.4 ± 0.82 c	1.5 ± 0.23 c
*P*_(PC)_	*	*	*	*	*
**Grape cultivar (GC)**					
Khalili (K)	30.3 ± 4.1 a	21.7 ± 4.2 a	1.23 ± 0.08 a	26.03 ± 1.4 b	1.96 ± 0.8 a
Perlette (P)	32.6 ± 2.5 b	32.6 ± 2.5 b	32.6 ± 2.5 b	32.6 ± 2.5 b	3.26 ± 0.25 b
*P*_(GC)_	*	*	*	*	*
**Temperature treatment (TT)**					
+ 24 °C (Normal; N)	27.2 ± 3.7 b	22.3 ± 0.6 a	1.23 ± 0.1 a	24.5 ± 0.19 b	2.02 ± 0.19 b
+ 2 °C (Stress; S)	30.9 ± 0.6 a	18.6 ± 0.61 b	1.05 ± 0 b	29.7 ± 0.89a	1.9 ± 0.93 b
*P*_(TT)_	*	*	*	*	ns
**P concentration × Cultivar × Temperature**					
0 × K × N	29.3 ± 0.39bc	20.9 ± 0.55 cd	0.62 ± 0.07d	26.8 ± 0.23 cd	2.6 ± 0.06a
0 × K × S	23.1 ± 0.36de	14.6 ± 0.21 ef	0.53 ± 0.1de	33 ± 1.19 ab	2.4 ± 0.05a
0.5 × K × N	33.8 ± 1.08a	25.3 ± 1.08ab	1.59 ± 0ab	31.3 ± 0.33b	1.76 ± 0.03ab
0.5 × K × S	30.5 ± 0.2bc	21.5 ± 0.06 cd	1.36 ± 0bc	35.5 ± 1.09 a	1.74 ± 0.05b
1.0 × K × N	35.2 ± 1.1 a	26.7 ± 1.15a	1.83 ± 0.03b	18.6 ± 0.10 f	1.58 ± 0.12bc
1.0 × K × S	30.1 ± 0.6b	21.4 ± 0.53 cd	1.46 ± 0.38b	24.1 ± 0.31 de	1.54 ± 0.05bc
0 × P × N	24.9 ± 0.23d	16.5 ± 0.20e	0.32 ± 0 ef	26 ± 0.5 cd	2.7 ± 0.04a
0 × P × S	21.7 ± 0.51 e	13.2 ± 0.62f	0.27 ± 0 f	31.1 ± 0.41 b	2.8 ± 0.08a
0.5 × P × N	32.1 ± 0.30ab	23.2 ± 0.45bc	1.46 ± 0.3 b	27.5 ± 0.6 c	1.79 ± 0.02bc
0.5 × P × S	29.3 ± 0.24bc	21 ± 0.20 cd	1.20 ± 0.03c	32.7 ± 0.49ab	1.8 ± 0.05bc
1.0 × P × N	30.3 ± 0.50bc	21.4 ± 0.28 cd	1.52 ± 0.04b	16.7 ± 0.83 f	1.56 ± 0.13bc
1.0 × P × S	28.4 ± 0.45c	19.9 ± 0.33d	1.49 ± 0.05b	22.1 ± 0.38e	1.39 ± 0.07c
*P*_(PC × GC)_	ns	ns	ns	ns	ns
*P*_(PC × TT)_	ns	ns	ns	ns	ns
*P*_(GC × TT)_	ns	ns	ns	ns	ns
*P*_(PC × GC × TT)_	ns	ns	ns	ns	ns

Mean values marked with the different letters are significantly different (*p* ≤ 0.05) using Duncan’s multiple range. Means ± SE (*N* = 3)

### Leaf micronutrients

The contents of Fe, Zn, and Cu were significantly influenced by the main effects of P concentration, cultivar, and temperature (*p* ≤ 0.01). The total Fe content was also significantly affected by the main effects of P concentration (*p* ≤ 0.05) and cultivar (*p* ≤ 0.01). Both ‘Khalili’ and ‘Perlette’ cultivars showed the highest total Fe and active Fe content with 0 mM P concentration and + 24 °C (Table 3). The lowest total Fe and active Fe content were observed in both ‘Khalili’ and ‘Perlette’ cultivars at 0.5 mM and 1 mM P concentrations under both temperature treatments. The Cu concentration was highest in both cultivars at a 0.5 mM P concentration under + 24 °C, while the lowest concentration was recorded at 1 mM with mM P concentrations under + 2 °C (Table 3). The highest Zn concentration was observed without P nutrition under + 2 °C in the ‘Khalili’ cultivar. In comparison, the lowest concentration was recorded with 1 mM P concentration under + 24 °C in the ‘Perlette’ cultivar (Table 3).

**Table 3:** The interaction effect of phosphorus (P; 0, 0.5, 1.0 mM) and temperatures (+ 2 and + 24 °C) on some micronutrient content of leaves in two grape cultivars differing in cold tolerance

Treatment	Copper (mg/kg)	Zinc (mg/kg)	Total iron (mg/kg)	Active iron (mg/kg)
**Phosphorus concentration (PC; mM)**				
0	4.06 ± 1.70 b	23.9 ± 0.83 a	83.2 ± 1.8 a	40.4 ± 1.02 a
0.5	8.9 ± 0.25 a	20.6 ± 0.55 b	65.3 ± 1.3 b	31.6 ± 1.11 b
1.0	4.49 ± 0.4 b	12.6 ± 0.44 c	35.7 ± 1.4 c	13.4 ± 1.05 c
*P*_(PC)_	*	*	*	*
**Grape cultivar (GC)**				
Khalili (K)	6.1 ± 0.46 a	18.4 ± 0.99 a	57.4 ± 1.4 b	29.3 ± 0.81 a
Perlette (P)	5.4 ± 0.40 a	19.7 ± 0.19 a	64.6 ± 1.3 a	28.6 ± 0.64 a
*P*_(GC)_	ns	ns	*	ns
**Temperature treatment (TT)**				
+ 24 °C (Normal; N)	6.6 ± 0.39 a	17.1 ± 0.19 b	67.4 ± 2.3 a	29.5 ± 0.93 a
+ 2 °C (Stress; S)	5.0 ± 0.42 b	21.06 ± 0.19 a	55.3 ± 1.5 b	28.3 ± 0.75 a
*P*_(TT)_	*	*	*	ns
**P concentration × Cultivar × Temperature**				
0 × K × N	4.5 ± 0.68de	21.6 ± 0.4 bcd	84.6 ± 3.4 a	42.9 ± 1.92 ab
0 × K × S	4.2 ± 0.28 de	28.2 ± 0.33 a	78.5 ± 3.2 ab	40.3 ± 0.98 b
0.5 × K × N	10.3 ± 0.40 a	18.7 ± 0.16 d	33.1 ± 3.7 e	30.5 ± 1.25 c
0.5 × K × S	8.6 ± 0.19 ab	23.2 ± 0.66 bc	24.6 ± 2.6 f	33.4 ± 1.32 c
1.0 × K × N	6.1 ± 0.63 cd	12.3 ± 0.60 ef	72.4 ± 3.4 b	14.7 ± 0.85 d
1.0 × K × S	3.2 ± 0.25 e	14.1 ± 0.81 e	52.2 ± 1.2 d	14.4 ± 0.91 d
0 × P × N	4.4 ± 0.36 de	21 ± 0.72 cd	86.4 ± 2.6 a	46.5 ± 1.76 a
0 × P × S	3.06 ± 0.27 e	25 ± 1.2 ab	83.9 ± 2.9 a	40.4 ± 0.72 b
0.5 × P × N	8.8 ± 0.41 ab	20.5 ± 0.04 c	52.6 ± 1.4 d	31.2 ± 1.12 c
0.5 × P × S	7.8 ± 2.5 bc	21.7 ± 0.4 bcd	30.8 ± 2.1 e	31.9 ± 0.97 c
1.0 × P × N	5.5 ± 0.37 d	10.2 ± 0.27 f	73.3 ± 2.7 b	15.5 ± 1.17 d
1.0 × P × S	3.03 ± 2.6 e	14.0 ± 1.01 e	61.8 ± 2.5 c	10.3 ± 0.70 e
*P*_(PC × GC)_	ns	ns	ns	ns
*P*_(PC × TT)_	ns	ns	ns	ns
*P*_(GC × TT)_	ns	ns	ns	ns
*P*_(PC × GC × TT)_	ns	ns	ns	ns

Mean values marked with the different letters are significantly different (*p* ≤ 0.05) using Duncan’s multiple range. Means ± SE (*N* = 3)

## Discussion

Phosphorus is an essential element for plant growth and development, playing a critical role in enhancing resistance to abiotic stresses, including frost. Studies have shown that optimizing P application in plants can reduce the negative effects of low-temperature stress by increasing nutrient uptake and enhancing antioxidant performance, thereby improving growth and yield under cold conditions [58, 61]. In wheat, P application, particularly under low-temperature stress, increased grain weight and reduced frost-induced damage [60]. These findings suggest the potential of P to improve cold resistance in other crops, particularly in temperate regions where frost can have significant negative impacts on crop performance. One of the major stressors threatening vineyards in the temperate areas is late spring frost [13].

Under cold stress (2 °C), chlorophyll content decreased in all vines compared with 24 °C. Interestingly, vines treated with 0.5 mM P showed higher chlorophyll content compared with vines treated with P at 1 mM, indicating the concentration-dependent effect of P on chlorophyll content under low temperature. These results are consistent with previous findings in wheat and *Arabidopsis*, indicating the simultaneous impact of nutritional and environmental stresses on chlorophyll content and photosynthetic performance [14, 52]. Our findings emphasize the importance of the nutritional role of phosphorus on changes in non-stomatal factors affecting photosynthesis (chlorophyll content) and continued growth and resilience under cold stress conditions.

A significant decrease in leaf RWC was observed with decreasing temperature, with a more pronounced reduction in the ‘Perlette’ compared with ‘Khalili’ cultivar. The highest RWC was recorded at 0.5 mM P and 24 °C, while no significant difference was observed between 0 and 1 mM P concentrations. These findings are consistent with previous studies. For example, [55] reported that P deficiency under low-temperature conditions exacerbates stress and reduces RWC in soybean. Similarly, [20] found that adequate P helps maintain RWC under drought stress in common bean. Cold stress initially affects the fluidity of cellular membranes, leading to water movement into the apoplast and the formation of ice crystals, which ultimately reduces RWC in plant tissues [7]. Phosphorus plays a protective role under cold stress. The 0.5 mM P treatment at 24 °C resulted in the highest RWC, while P deficiency, especially under low temperatures, intensified stress and further decreased RWC. Therefore, P application can be considered an effective strategy to support water status in plants subjected to cold stress.

In this study, it was observed that the concentrations of H_2_O_2_ and MDA increased with decreasing temperature across all three P levels. These values were higher in the ‘Perlette’ cultivar compared with the ‘Khalili’ cultivar. An increase in H_2_O_2_ and MDA concentrations was also recorded at 0 and 1mM P concentrations even under non-stress (optimal temperature) conditions, suggesting that these two P levels may be perceived by the plant as a form of nutritional stress.

In line with our result, P application in alfalfa significantly decreased the levels of H_2_O_2_ and MDA under low-temperature conditions while enhancing the activity of antioxidant enzymes such as SOD and CAT [62]. Similarly, foliar application of KH_2_PO_4_ in wheat flag leaves under spring cold stress reduced the concentrations of H_2_O_2_ and MDA and improved physiological parameters, particularly antioxidant enzyme activities [9]. These results confirm that P can play a critical role in alleviating oxidative stress and enhancing plant tolerance to low temperatures. Considering the structural role of P in cell membrane phospholipids and cellular energy-supplying molecules such as ATP, it seems that this element regulates the movement of substances into and out of cells by maintaining the membrane structure [35]. In the present study, this role of P was confirmed by reducing ion leakage and lower production of MDA and H_2_O_2_ in treated vines leaves under low temperature stress specially at 0.5 mM concentration.

The present study demonstrated that simultaneous reductions in P concentration and temperature led to a significant increase in anthocyanin content in two cultivars, ‘Khalili’ and ‘Perlette’, with the highest anthocyanin levels observed at 0.5 mM P and 2 °C. These findings are consistent with previous research; for instance, studies on lettuce have reported that P deficiency combined with decreased root-zone temperature enhances anthocyanin accumulation [26]. Moreover, molecular mechanisms explored in *Arabidopsis thaliana* underscore the regulatory role of P in anthocyanin biosynthesis pathways [40]. Overall, P limitation and low temperature act as environmental stressors that activate anthocyanin biosynthetic pathways, serving as protective mechanisms in plants. However, in agreement with earlier reports, high P concentrations (1 mM) suppressed anthocyanin production, likely due to P toxicity, a factor that has been less addressed in prior studies. Therefore, the current results corroborate the interactive effects of P availability and temperature in modulating anthocyanin synthesis, advancing our understanding of plant adaptive responses to environmental stress.

In the present study, although low P concentration and reduced temperature increased antioxidant enzyme activity in both ‘Khalili’ and ‘Perlette’ cultivars, enzyme activity sharply decreased at 1 mM P under temperature treatments (+ 2 °C and + 24 °C). This response in grape seedlings may be attributed to a severe reduction in the levels of essential micronutrients such as Zn, Cu, and Fe at this P concentration. The presence of these metal elements is crucial for the synthesis and activity of antioxidant enzymes, especially those containing heme groups. These findings align with those of [44], who demonstrated that P deficiency in *Citrus grandis* leads to increased activities of antioxidant enzymes such as SOD, CAT, and APX, indicating enhanced oxidative stress responses under low P conditions. Similarly, [64] reported increased antioxidant enzyme activities in wild grape species subjected to cold stress, consistent with the observed temperature-induced increase in enzyme activity in this study. Furthermore, [19] showed that P deficiency triggers antioxidant defense mechanisms through enhanced enzyme activities and accumulation of non-enzymatic antioxidants in plants, supporting the notion that low P stimulates antioxidant responses. However, the marked decline in enzyme activity at the high P concentration (1 mM) observed in this study, likely due to micronutrient imbalances affecting Zn, Cu, and Fe availability, provides a novel insight less explored in previous research, highlighting the importance of micronutrient interactions in regulating antioxidant enzyme functionality.

In the present study, application of 0.5 mM P under + 2 °C conditions resulted in a significant increase in polyamine content compared with + 24 °C in both ‘Khalili’ and ‘Perlette’ grape cultivars. Interestingly, elevated polyamine levels were also detected at 0 and 1 mM concentrations under + 24 °C, indicating that their accumulation is not exclusively triggered by cold stress. These findings are consistent with previous studies reporting enhanced polyamine accumulation under a variety of abiotic stresses, including heavy metal toxicity, salinity, drought, and low temperature [8]. Polyamines are recognized as key regulators in plant responses to biotic and abiotic stresses. They contribute to stress tolerance by modulating the accumulation of soluble sugars, proline, and specific amino acids. Furthermore, polyamines are involved in vital physiological and developmental processes such as cell growth, differentiation, metabolism, and senescence, largely through interactions with plant hormones [1]. Functioning as hormone-like compounds, polyamines contribute to cellular homeostasis by binding to anionic and cationic cellular components, thereby mitigating oxidative stress, photooxidation, and lipid peroxidation. These actions ultimately support the structural integrity and stability of plant cells [18].

At a P concentration of 0 mM and a temperature of + 2 °C, the highest levels of sucrose, fructose, glucose, and starch were observed, whereas the lowest levels of these sugars and starch were recorded at a P concentration of 1 mM. Under cold stress conditions, the amount of soluble sugars in the leaves of grape seedlings significantly increased compared with plants without cold stress. This increase was accompanied by a decrease in P concentration. Although the levels of soluble sugars increased, they significantly decreased at a P concentration of 1 mM. Similar results have been reported in tobacco, where under cold stress, the levels of soluble sugars, such as sucrose, glucose, and fructose, increased in tobacco leaves. Additionally, starch levels decreased in plants under cold stress. The addition of P specifically altered the interaction between temperature and P levels. Under low-temperature conditions, sucrose and glucose levels increased in plants with optimum P concentrations, while starch levels decreased [63]. These results suggest that P can positively affect the metabolism of sugars and starch under cold stress, thereby enhancing the plant’s resistance.

In our experiment, Ca content generally increased in response to cold stress across all P concentrations tested. However, under a temperature of + 24 °C without P supplementation, Ca content decreased, with a more pronounced reduction observed at 1 mM P compared with 0 mM. Under cold stress conditions, both Ca and Zn levels significantly increased, whereas N, K, P, and Cu concentrations declined. Although Fe content decreased at 0.5 mM P relative to 0 mM, it increased again at 1 mM P; nonetheless, symptoms of Fe deficiency were evident in the plants. Physiological and morphological assessments revealed that while Fe increased with elevated P supply, the concentration of active Fe markedly declined. P can inhibit the activity of this essential element by modulating genes involved in Fe regulation and binding directly to Fe [51]. Measuring active Fe is therefore critical for distinguishing between true and apparent Fe deficiencies, especially under conditions promoting Fe deposition in plant tissues [50].

Previous studies have reported increased levels of Mn, Fe, and Cu alongside decreases in Ca and Mg under P-deficient conditions [53]. Conversely, elevated P availability has been associated with reductions in Fe, Ca, Zn, and Cu concentrations [4]. Our findings largely corroborate these reports, particularly the increases in Fe and Cu under P deficiency and decreases in Ca and other minerals at high P levels. Notably, the observed increase in Ca under cold stress in our study contrasts with some prior findings, suggesting that the interaction between P nutrition and environmental stressors such as temperature can differentially influence mineral nutrient dynamics. These results underscore the complexity of nutrient interactions under combined stress conditions and highlight the need to consider both nutritional and environmental factors when evaluating mineral uptake and plant stress responses.

According to studies in this area, protein levels increased with decreasing temperature, but at a P concentration of 1 mM, no significant difference was observed between the + 2 °C and + 24 °C temperature treatments. The P concentration at 1 mM may be recognized by the plant as a nutritional stress, leading to increased ROS, DNA degradation, and inhibited protein synthesis.

With increasing P concentration and decreasing temperature, the abscisic acid (ABA) level increased, so that in both ‘Khalili’ and ‘Perlette’ cultivars, the highest ABA concentration was observed at 1 mM P under 2 °C, while the lowest concentration of this hormone was recorded at 0.5 mM P under 24 °C in both cultivars. Although there are no reports on the effect of P under cold conditions on ABA, a similar study showed that exogenous application of abscisic acid could improve plant tolerance to P deficiency and enhance antioxidant activities and P utilization under P-limited conditions [34].

## Conclusion

According to the results of this study, the use of 0.5 mM P concentration showed the highest growth indices and tolerance to winter cold stress. In P-free treatments, in the absence of cold stress, the plants exhibited growth stagnation with dark and brittle leaves, showing low resistance to cold stress. However, the resistance of this group of plants was much higher than that under the 1 mM P treatment. At 1 mM P concentration, although the plants did not experience growth stagnation, a significant decrease in vegetative growth quality indices and leaf chlorosis was observed. These plants showed the lowest cold resistance indices under + 2 °C stress. Complete death of the seedlings after cold stress was observed in both ‘Perlette’ and ‘Khalili’ cultivars at 1 mM concentration. Finally, it was found that moderate P (0.5 mM) increases cold tolerance, while excessive P (1 mM) has a negative effect on the cold tolerance of grapes.

## Supplementary Information

Below is the link to the electronic supplementary material.

Supplementary Material 1

## Data Availability

The data that support the findings of this study are available from the co-corresponding authors upon reasonable request.
