# Supplementary material for: Phosphorus and cold stress: physiological and biochemical assessment in two grape (Vitis vinifera L.) cultivars
Source: Biol Res. 2026 Mar 14;59:24. doi: 10.1186/s40659-026-00683-0 (PMC13101383; doi:10.1186/s40659-026-00683-0)
Supplement: Supplementary file 1 — Supplementary Material 1 [file 40659_2026_683_MOESM1_ESM.docx]

Khadivi

Ali (CA)

Yazgan

Tunç

**BRES-D-25-00767**

Salehi-Arjmand (CA)

Hossein

Mousa

Rasouli

Karimi

Rouhollah

Maryam

Zaeri-Behrooz

**Phosphorus and cold stress: physiological and biochemical assessment in two grape (*Vitis vinifera* L.) cultivars**

Yazgan

Tunç

Khadivi (CA)

Ali

Karimi

Rouhollah

Zaeri-Behrooz

Maryam

We had a mistake to providing authors at the time of submitting the manuscript that it was corrected in the edited version with the consent of the authors

Maryam Zaeri Behrooz: Writing – original draft, Methodology, Investigation; Rouhollah Karimi: Writing – review & editing, Visualization, Validation, Supervision, Funding acquisition; Ali Khadivi: Writing – review & editing, Visualization, Resource; Methodology, Formal Analysis, Validation; Yazgan Tunç: Writing – review & editing, Visualization, Validation, Formal Analysis. All authors approved the final manuscript.

Not applicable

Not applicable


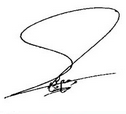

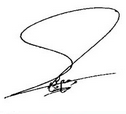

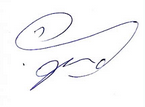

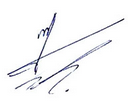

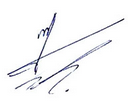


Khadivi (CA)

Tunç

09 February 2026

Ali

Yazgan

Zaeri-Behrooz

Rouhollah

Karimi

Maryam

Rasouli

Mousa

09 February 2026

09 February 2026

09 February 2026

09 February 2026
